# Slapping automatism in epileptic seizures: a case series

**DOI:** 10.3389/fnhum.2025.1593597

**Published:** 2025-08-25

**Authors:** Shenglin Hao, Ziyi Feng, Haifeng Zhao, Wei Liu, Shikun Zhan, Bomin Sun, Qiang Zhou, Chunyan Cao

**Affiliations:** ^1^Department of Neurosurgery, Affiliated Ruijin Hospital, Shanghai Jiao Tong University, School of Medicine, Shanghai, China; ^2^Department of Laboratory Medicine, Gongli Hospital, Shanghai, China

**Keywords:** slapping automatism, frontal lobe epilepsy, temporal lobe epilepsy, obsessive-compulsive disorder, stereo-electroencephalography (SEEG)

## Abstract

**Background:**

Slapping automatism is a type of automatism observed during epileptic seizures, but its underlying electrophysiological mechanisms remain poorly understood. Stereo-electroencephalography (SEEG) provides a unique opportunity to investigate the associated cortical areas with epileptiform discharges during the slapping automatism.

**Case report:**

We report five cases of drug-resistant epilepsy in which SEEG recordings captured slapping automatism. In four patients, slapping movements coincided with ictal electrical evolution in the prefrontal lobe, specifically involving the orbitofrontal cortex (OFC), lateral prefrontal cortex (PFC), and anterior cingulate (ACC). Among them, two cases were temporal lobe epilepsy (TLE), while the other two were frontal lobe epilepsy (FLE). Additionally, we describe one patient with TLE and obsessive-compulsive disorder (OCD), whose SEEG recordings revealed frequent interictal epileptiform discharges in both the medial temporal and prefrontal regions. The seizures originated in the temporal lobe and rapidly evolved into generalized seizures. Although slapping automatism was not observed during seizures, the patient exhibited repetitive patting on the bed during the interictal period. Based on SEEG-defined epileptic networks, all patients underwent surgical interventions, including lesion resection and/or disconnection procedures. At follow-up, all three patients achieved Engel class I outcomes and two patients Engel class II.

**Conclusion:**

SEEG recordings in this study confirm a strong association between orbitofrontal lobe epileptic activity and slapping automatism. Notably, we report a case of TLE with comorbid OCD, characterized by frequent epileptiform discharges in the orbitofrontal lobe, providing direct electrophysiological evidence of prefrontal abnormalities in OCD. Together, these findings highlight a shared pathological network of orbitofrontal-striatal-pallidal-thalamic between frontal and temporal epilepsy with slapping automatism and OCD.

## 1 Introduction

Epilepsy is a prevalent chronic neurological disorder caused by abnormal synchronous neuronal discharges in the brain. Its clinical manifestations are highly diverse, reflecting the wide range of brain regions involved. Among these manifestations, automatisms, repetitive, involuntary motor behaviors are frequently observed and constitute an important component of seizure semiology (Berg et al., 2010). One such behavior, repetitive slapping or patting, represents a distinct form of automatism that has been linked to frontal lobe epilepsy (Kotagal et al., 2003); however, the specific sublobar regions underlying these behaviors have not been thoroughly investigated. Extensive evidence indicates that the frontal lobe, particularly the medial prefrontal cortex (PFC), orbitofrontal cortex (OFC), and anterior cingulate (ACC), plays a pivotal role in motor planning, execution, and inhibition (Kringelbach & Rolls, 2004; Ridderinkhof et al., 2004). Notably, patients with frontal lobe epilepsy (FLE) may also exhibit ritualistic, obsessive-compulsive disorder (OCD)-like repetitive behaviors (Kaplan, 2010). Studies have shown that OCD is closely linked to dysfunction in the PFC and OFC (Aouizerate et al., 2004). Additionally, dysregulation of the orbitofrontal-striatal-pallidal-thalamic circuit has been strongly associated with ritualistic and repetitive behaviors. Previous studies have highlighted that up to 22% of patients with temporal lobe epilepsy (TLE) exhibit OCD, particularly traits such as ordering, washing, and checking, suggesting a preferential overlap between TLE and compulsivity-related psychopathology (Kaplan, 2010). Further, neuropsychological and neuroimaging studies have implicated dysfunction in fronto-striatal and orbitofrontal circuits as shared pathological substrates of both TLE and OCD (Paranhos et al., 2022). These overlapping networks raise the possibility that specific seizure-related automatisms, such as slapping, may emerge from aberrant activity within circuits also involved in compulsive behavior regulation. Stereoelectroencephalography (SEEG), which allows for precise mapping of seizure onset and propagation across cortical and subcortical structures, provides a powerful tool for elucidating the neural mechanisms underlying such automatisms.

In this study, we retrospectively reviewed all patients with drug-resistant focal epilepsy who underwent SEEG evaluation at Ruijin Hospital, Shanghai Jiao Tong University School of Medicine, between 2021 and 2025, and identified five cases with hand-related automatisms, including slapping, clapping, or patting, recorded during SEEG (Supplementary Table 1). Among these, two patients had frontal lobe epilepsy, two had temporal lobe epilepsy, and one had temporal epilepsy with comorbid OCD. Notably, this latter case demonstrated interictal spiking activity in the bilateral OFC and occasional right-hand patting, suggesting a potential mechanistic link between seizure-related automatisms and compulsive behaviors. Through comprehensive analyses of clinical presentations, neuroimaging findings, and SEEG recordings, we aim to investigate how epileptiform discharges in frontal-temporal regions propagate through orbitofrontal-subcortical networks to induce specific automatisms, and to explore potential links between these automatisms and OCD-related behaviors.

### 1.1 Case 1

The patient is a 37-year-old right-handed woman with a 37-year history of epilepsy. At 5 months of age, following hypoxia due to dehydration from diarrhea, the patient began experiencing absence seizures, with a frequency of approximately five times per day and lasting approximately 2–3 s. At age 34, seizure manifestations evolved to include loss of consciousness, a greeting-like vocalization, and automatisms such as slapping of the right hand or bilateral hand clapping, lasting 5–6 s and occurring almost daily. By age 36, the patient developed psychiatric symptoms, including auditory hallucinations and self-talking behaviors.

**Figure 1:**
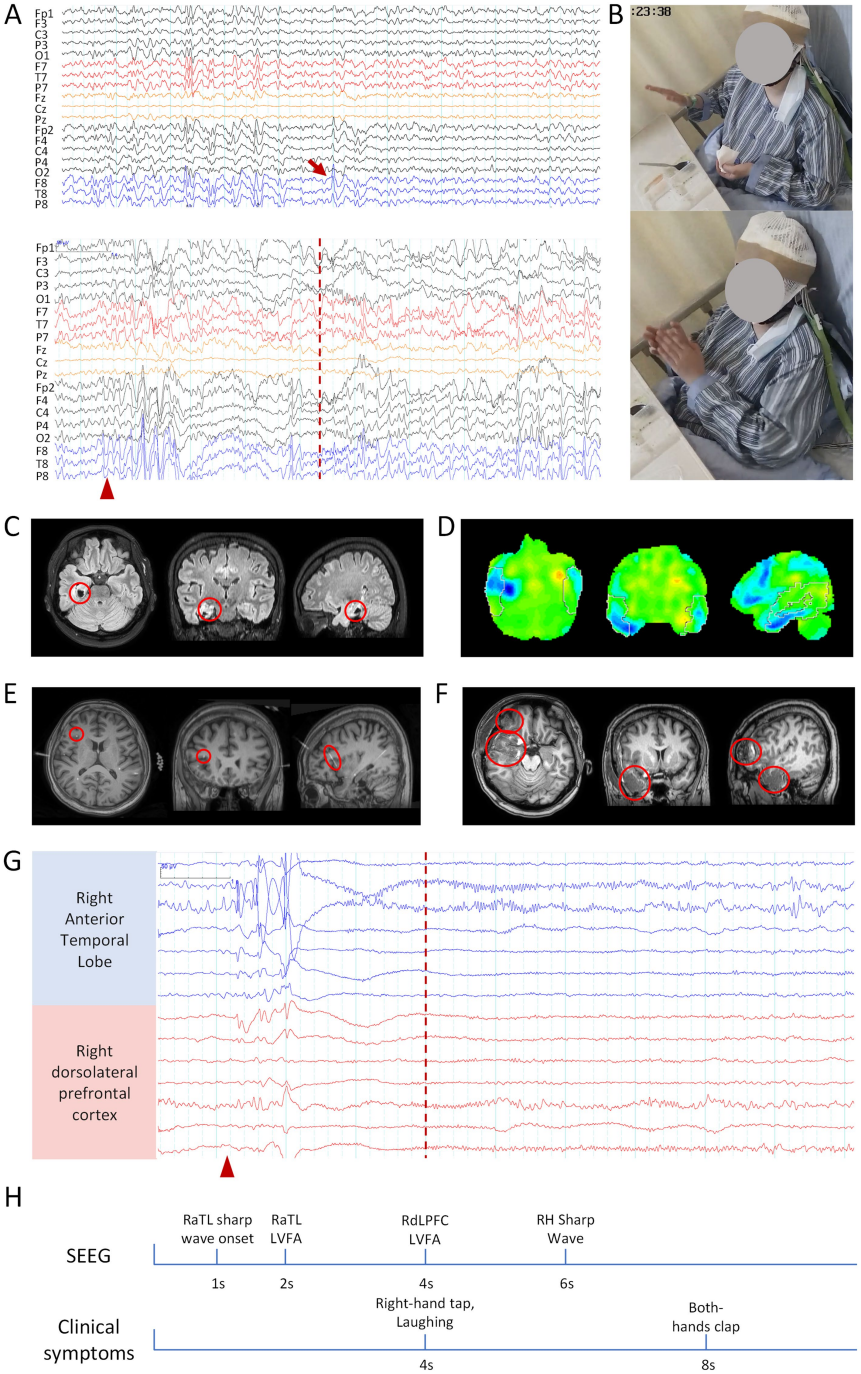
Clinical information related to patient 1. **(A)** Scalp EEG: the upper panel shows interictal EEG, and the lower panel shows ictal EEG. Bandpass filter: 0.5–30 Hz; Sensitivity: 10 μV/mm. **(B)** Hand-clapping automatism observed during the seizure. **(C)** MRI, right hippocampal atrophy. **(D)** 18F-FDG PET, Hypometabolism of Glucose in the right temporal lobe. **(E)** Coregistered preoperative MRI and post-implantation SEEG CT, with the red circle indicating the frontal electrode related to clapping. **(F)** Postoperative MRI, with the red circle marking the resected brain region. **(G)** Ictal SEEG, blue channels indicate SOZ, while red channels represent the propagation of epileptic discharges to the frontal lobe. The red arrow indicates interictal epileptic discharges, the red triangle marks the seizure onset, and the red dashed line represents the onset of the hand-clapping symptom. The scale marker is located in the upper left corner of the EEG. Bandpass filter: 0.5–30 Hz; Sensitivity: 100 μV/mm. **(H)** Timeline diagram of SEEG discharges and clinical symptoms. RaTL, right anterior temporal lobe; LVFA, low-voltage fast activity; RdLPFC, right dorsolateral prefrontal cortex; RH, right hippocampus.

Video EEG (VEEG) monitoring revealed interictal sharp wave discharges in the bilateral temporal regions and the right frontal lobe, with a predominant focus in the right temporal region (T8, F8). Ictal EEG recordings demonstrated bilateral temporal sharp waves, maximal over the right temporal lobe, with propagation to the right frontal lobe (Figures 1A,B). MRI revealed cystic degeneration involving the right hippocampus (Figure 1C), and 18F-FDG PET showed reduced metabolism in the right temporal lobe and right prefrontal lobe (Figure 1D).

Based on these findings, a preliminary diagnosis of right TLE was established. Subsequent SEEG evaluation identified interictal rhythmic phase inversion in the right anterior temporal lobe, hippocampus-amygdala complex, and anterior insular cortex, along with persistent slow-wave discharges in the left frontal lobe. Ictal SEEG recordings demonstrated seizure onset in the right anterior temporal lobe, with propagation to the right dorsolateral prefrontal cortex (dlPFC) within 3 s, coinciding with the emergence of right slapping and then clapping automatism (Figures 1E,G,H).

The patient underwent a right anterior temporal lobectomy, including resection of the anterior insula, and frontal operculum cortex, along with SEEG-guided RF-TC of the right insula (Figure 1F). At the 16-month postoperative follow-up, the patient achieved an Engel class I seizure outcome.

### 1.2 Case 2

The patient is a 38-year-old right-handed woman with a 32-year history of epilepsy and comorbid severe anxiety and depression (Supplementary Table 2). The first generalized tonic–clonic seizure (GTCS) occurred at age 6 following a febrile episode. The current seizure manifestations include two distinct types: one characterized by brief staring episodes with loss of awareness lasting 1–2 min, often preceded by a sensation of palpitations; the other involving loss of awareness accompanied by right leg stomping and right-hand slapping, sometimes leading to urinary incontinence, lasting approximately 3 min. Seizures occur 4–5 times per month.

**Figure 2:**
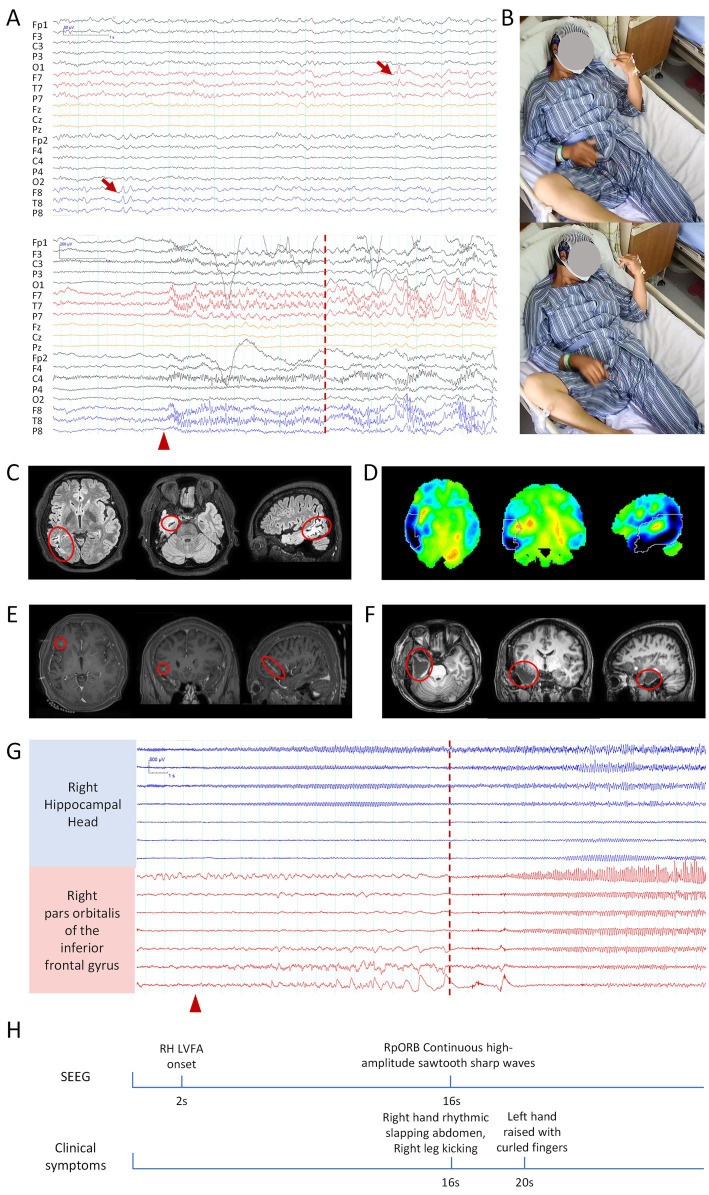
Clinical information related to patient 2. **(A)** Scalp EEG: the upper panel shows interictal EEG, and the lower panel shows ictal EEG. Bandpass filter: 0.5–30 Hz; Sensitivity: 10 μV/mm. **(B)** Hand-clapping automatism observed during the seizure. **(C)** MRI, Patchy hyperintense lesions in the right temporo-occipital white matter, with mild atrophy of the right hippocampus. **(D)** 18F-FDG PET, hypometabolism of glucose in the right temporal lobe. **(E)** Coregistered preoperative MRI and post-implantation SEEG CT, with the red circle indicating the frontal electrode related to clapping. **(F)** Postoperative MRI, with the red circle marking the resected brain region. **(G)** Ictal SEEG, blue channels indicate SOZ, while red channels represent the propagation of epileptic discharges to the frontal lobe. The red arrow indicates interictal epileptic discharges, the red triangle marks the seizure onset, and the red dashed line represents the onset of the hand-clapping symptom. The scale marker is located in the upper left corner of the EEG. Bandpass filter: 0.5–30 Hz; Sensitivity: 70 μV/mm. **(H)** Timeline diagram of SEEG discharges and clinical symptoms. RH, right hippocampus; LVFA, low-voltage fast activity; RpORB, right pars orbitalis of the inferior frontal gyrus.

VEEG monitoring showed interictal sharp wave discharges in the bilateral temporal and frontal lobes, predominantly in the left temporal region (FT7, T7). Ictal EEG revealed fast rhythmic epileptiform discharges originating from both temporal lobes (Figures 2A,B). MRI demonstrated patchy hyperintense in the right temporo-occipital white matter and mild hippocampal atrophy on the right (Figure 2C), while 18F-FDG PET indicated markedly reduced metabolism in the right temporo-occipital lobe (Figure 2D).

Based on a comprehensive evaluation, the patient was diagnosed with temporal lobe epilepsy. It was hypothesized that the epileptic focus originated in the right temporal lobe and subsequently propagated to the left temporal lobe. Due to extensive atrophy in the right temporal lobe, epileptiform activity in the right hemisphere was less pronounced on scalp EEG compared to the left. To further localize the epileptogenic network, SEEG electrodes were implanted. Interictal SEEG revealed delta waves in the left hippocampal head and small sharp wave discharges in the right temporo-parieto-occipital junction and left sensorimotor cortex. Ictal SEEG recordings demonstrated seizure onset in the right hippocampal head, with propagation to the right pars orbitalis of the inferior frontal gyrus 14 s later, coinciding with the onset of slapping automatism (Figures 2E,G,H).

The patient subsequently underwent a standard right anterior temporal lobectomy, including resection of the epileptogenic focus in the orbitofrontal gyrus and the temporo-parieto-occipital junction, as well as SEEG-guided RF-TC of the left hippocampal head, and left orbitofrontal gyrus (Figure 2F). At the 9-month postoperative follow-up, the patient achieved an Engel class I seizure outcome. In addition, the patient’s preoperative severe depression and significant anxiety improved postoperatively, with follow-up assessments showing mild depression and mild anxiety (Supplementary Table 2).

### 1.3 Case 3

The patient is a 20-year-old right-handed man with an 11-year history of epilepsy. The first GTCS occurred at age 9, lasting approximately 30 s. At age 20, seizures increased in frequency, occurring 2–3 times per month and lasting 3–5 s. Current seizure manifestations include loss of awareness, right hand slapping, and generalized convulsions. Mild obsessive-compulsive symptoms and mild anxiety were reported by the family.

**Figure 3:**
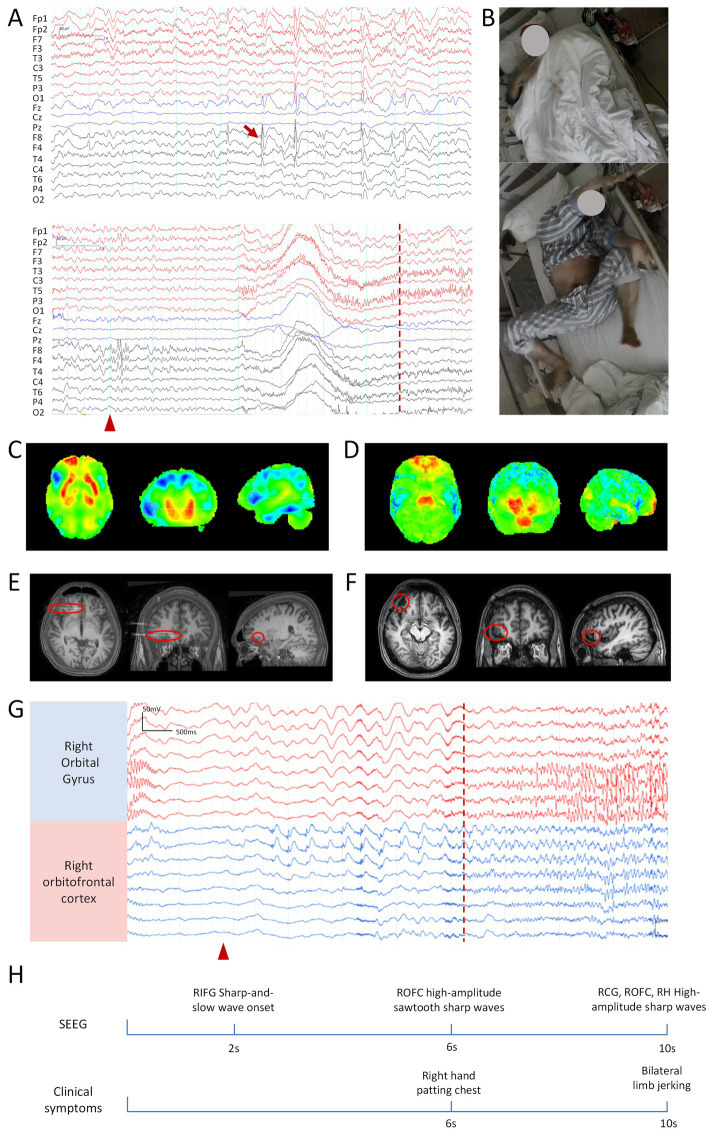
Clinical information related to patient 3. **(A)** Scalp EEG: the upper panel shows interictal EEG, and the lower panel shows ictal EEG. Bandpass filter: 0.5–30 Hz; Sensitivity: 10 μV/mm. **(B)** Hand-clapping automatism observed during the seizure. **(C)** PET, hypometabolism in the right frontal lobe. **(D)** 18F-FDG PET, hypometabolism of glucose in the right temporal lobe. **(E)** Coregistered preoperative MRI and post-implantation SEEG CT, with the red circle indicating the frontal electrode related to clapping. **(F)** Postoperative MRI, with the red circle marking the resected brain region. **(G)** Ictal SEEG, blue channels indicate SOZ, while red channels represent the propagation of epileptic discharges to the frontal lobe. The red arrow indicates interictal epileptic discharges, the red triangle marks the seizure onset, and the red dashed line represents the onset of the hand-clapping symptom. The scale marker is located in the upper left corner of the EEG. Bandpass filter: 0.5–70 Hz; Sensitivity: 70 μV/mm. **(H)** Timeline diagram of SEEG discharges and clinical symptoms. RIFG, right inferior frontal gyrus; ROFC, right orbitofrontal cortex; RCG, right cingulate gyrus; RH, right hippocampus.

VEEG showed interictal sharp wave discharges in the bilateral frontal lobes (F3, F7, F4, F8). Ictal EEG revealed high-amplitude transient sharp waves in the right frontal and temporal lobes (F8, F4, T4, T6), followed after 3 s by generalized electromyographic artifacts, predominantly in the bilateral temporal regions (Figures 3A,B). MRI showed no significant abnormalities, while 18F-FDG PET indicated reduced metabolism in the right frontal and right temporal lobes (Figures 3C,D).

Based on these noninvasive findings, a preliminary diagnosis of FLE was made, with possible temporal involvement. The patient subsequently underwent SEEG monitoring. Interictal SEEG showed epileptiform discharges in the right hippocampus, orbitofrontal gyrus, anterior insular cortex, and frontal lobe. Ictal SEEG demonstrated seizure onset in the right inferior frontal gyrus, with propagation to the right OFC after 4 s, coinciding with the onset of slapping automatism (Figures 3E,G,H).

The patient underwent resection of the right orbitofrontal gyrus and anterior insula, along with SEEG-guided RF-TC targeting the bilateral frontal lobe and right orbitofrontal gyrus (Figure 3F). At the 50-month postoperative follow-up, the patient achieved an Engel class II seizure outcome. The patient exhibited no obsessive-compulsive or anxiety symptoms after surgery (Supplementary Table 2).

### 1.4 Case 4

The patient is a 12-year-old right-handed girl with a 2-year history of epilepsy, experiencing more than 10 seizures per month. She exhibited three seizure types: absence seizures characterized by brief staring and impaired awareness; hand clapping seizures and shouting, and generalized tonic–clonic seizures. The hand clapping seizures were characterized by shouting, bilateral hand clapping, accompanied by bilateral leg stomping and impaired awareness, lasting approximately 1 min. The hand clapping seizures were the most frequent seizure type in this patient.

VEEG showed interictal sharp wave discharges in the bilateral frontal lobes and left temporal lobe, with a predominant focus in the left frontal lobe (AF7, F7). Ictal EEG recordings revealed epileptiform discharges in the left frontal and temporal lobes, which propagated to the contralateral side within 2 s. The seizures began with a panic emotion, followed by shouting, bilateral hand clapping, bilateral knee flexion, and impaired awareness (Supplementary Figures 1A,B). MRI findings were consistent with Tuberous Sclerosis Complex (TSC), showing several patchy hyperintensities in the bilateral frontoparietal lobes (Supplementary Figure 1C). 18F-FDG PET revealed reduced metabolism in the left frontal lobe (Supplementary Figure 1D).

Based on this comprehensive evaluation, the patient was diagnosed with frontal lobe epilepsy. SEEG was subsequently performed, revealing interictal slow-wave in the left anterior PFC and orbitofrontal gyrus, as well as fast rhythmic discharges in the right superior parietal lobule. Ictal SEEG recordings localized seizure onset to the left anterior PFC, with propagation to the left dorsal ACC after 7 s. Both regions exhibited discharge evolution after 9 s when the clapping occurred (Supplementary Figures 1E,G,H).

The patient underwent resection of the epileptic focus in the left frontal lobe, along with SEEG-guided RF-TC of the bilateral cingulate gyrus, left orbitofrontal gyrus, and left ventromedial PFC (Supplementary Figure 1F). At the 15-month postoperative follow-up, the patient remained seizure-free and was classified as Engel class I.

### 1.5 Case 5

The patient is a 49-year-old right-handed woman with a 47-year history of epilepsy, a 20-year history of mania, and a 1-year history of OCD. At age 2, following a febrile convulsion, the patient began experiencing bilateral upper limb spasms lasting 1–2 min, occurring 2–3 times per week. At age 29, the patient exhibited manic symptoms, including aggression. Current seizure manifestations include altered awareness, a fixed gaze, and limb spasms and GTCS. Over the past year, the patient developed compulsive behaviors, characterized by excessive laundry washing, sweeping, and intermittent clapping.

VEEG revealed interictal sharp wave discharges in the bilateral frontotemporal regions, predominantly in the left temporal lobe (F7, T3). Ictal EEG demonstrated independent seizure onsets in both temporal lobes (Supplementary Figures 2A,B). MRI showed scattered small patchy hyperintensities in the bilateral frontoparietal lobes and atrophy of the bilateral hippocampi (Supplementary Figure 2C). 18F-FDG PET revealed hypometabolism in the bilateral frontal and parietal lobes (Supplementary Figure 2D).

Based on these multimodal findings, the patient was diagnosed with TLE and temporal-plus epilepsy syndrome. SEEG monitoring revealed interictal rhythmic high-amplitude discharges in both hippocampi, persistent sharp discharges in the right operculum and orbitofrontal gyrus, and intermittent sharp discharges in the left operculum. Ictal SEEG recordings localized seizure onset to the bilateral hippocampal head, with propagation to the bilateral pars orbitalis of the inferior frontal gyrus after a few seconds, followed by the onset of GTCS. Notably, right hand patting behaviors were observed during interictal recordings, corresponded with epileptiform discharges in the right operculum and orbitofrontal gyrus (Supplementary Figures 2E,G,H). During the course of VEEG and SEEG monitoring, the patient experienced five left temporal seizures and two right temporal seizures.

Given the presence of prominent psychiatric symptoms, severely impaired cognitive function, and a lack of caregiving support, a combined surgical strategy was adopted. The patient underwent an anterior capsulotomy to address OCD symptoms and a standard left anterior temporal lobectomy for epilepsy control, along with SEEG-guided RF-TC targeting the right hippocampal head, right anterior insular cortex, and right orbitofrontal gyrus (Supplementary Figure 2F). At the 6-month postoperative follow-up, the patient achieved an Engel class II seizure outcome. Compulsive OCD behaviors, including repetitive washing disappeared after surgery.

## 2 Discussion

This case series provides rare and compelling electrophysiological evidence linking slapping automatism to frontal lobe epileptic networks, with particular involvement of the Pars Orbitalis and dlPFC. Through direct SEEG recordings, we demonstrate that slapping or clapping behaviors during seizures are tightly coupled with ictal propagation to prefrontal subregions, regardless of the initial seizure onset zone (temporal vs. frontal). Furthermore, our findings highlight the intricate interplay between slapping automatism and compulsive behaviors, and suggest overlapping neurocircuitry in patients with FLE, TLE plus and comorbid OCD.

### 2.1 Slapping automatism: a marker of prefrontal involvement

**Figure 4:**
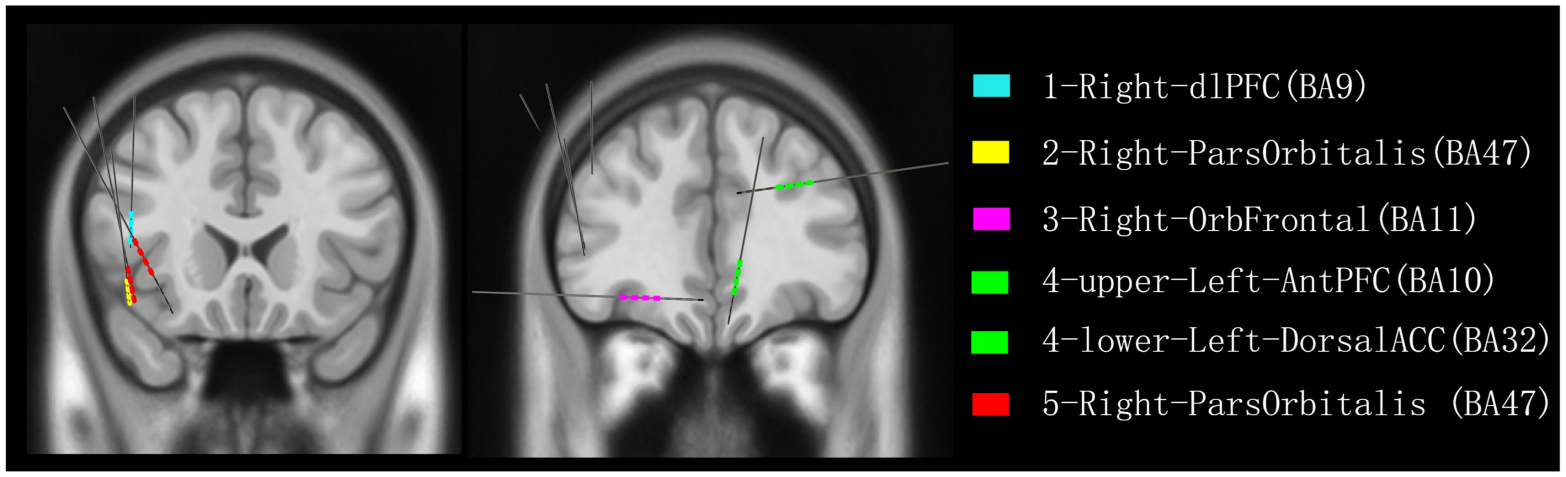
Reconstruction of all recorded contacts detecting frontal lobe discharges associated with slapping or patting in five patients. Color coding indicates the discharges recorded from each patient: blue for patient 1, yellow for patient 2, pink for patient 3, green for patient 4, and red for patient 5. dlPFC, dorsolateral prefrontal cortex; ParsOrbitalis, pars orbitalis of the inferior frontal gyrus; OrbFrontal, orbitofrontal cortex; AntPFC, anterior prefrontal cortex; DorsalACC, dorsal anterior cingulate cortex.

In all four patients with documented slapping or clapping automatisms during seizures (Cases 1–4), SEEG revealed ictal propagation to frontal subregions at or just prior to the appearance of the motor behavior. Notably, the OFC, dlPFC, and ACC were consistently involved (Figure 4). These regions are known for their roles in motor execution, emotional modulation, and inhibitory control (Aouizerate et al., 2004)—functions that may become dysregulated during seizure activity, resulting in stereotyped, semi-purposeful behaviors such as slapping.

Our data support previous observations that motor automatisms, especially complex bilateral behaviors like clapping or slapping, are more often associated with FLE, particularly in the orbitofrontal and mesial frontal circuits (Fohlen et al., 2004; Waterman et al., 1987; Schönecker et al., 2019). Importantly, two patients in our series (Cases 1 and 2) were ultimately diagnosed with TLE but demonstrated clear frontal involvement during the automatisms, consistent with the “temporal-plus” epilepsy concept (Kahane et al., 2015), Which refers to temporal lobe epilepsy with early and consistent seizure propagation to neighboring extratemporal regions, such as frontal area, particularly when propagation occurs rapidly through frontotemporal networks.

### 2.2 Frontotemporal networks and psychiatric comorbidity

The fifth case in our series further extends the significance of these findings by illustrating a convergence between epileptic networks and compulsive behaviors. This patient, diagnosed with TLE and comorbid OCD, demonstrated interictal patting behaviors—which coincided with epileptiform discharges in the orbitofrontal gyrus and opercular regions, areas heavily implicated in OCD pathophysiology (Aouizerate et al., 2004; Amo et al., 2004). Remarkably, the patient’s compulsive behaviors resolved following capsulotomy and epilepsy surgery, underscoring the shared neural substrate hypothesis of epilepsy and OCD.

Our findings are congruent with neuroimaging and lesion studies suggesting that fronto-striato-thalamic circuits, especially those involving the OFC and ACC, are central to compulsivity (Schönecker et al., 2019; Saxena and Rauch, 2000; Milad and Rauch, 2012; Menzies et al., 2007). In epileptic patients, chronic network dysfunction—manifested as both seizures and psychiatric symptoms—may be underpinned by abnormal oscillatory activity or pathological connectivity within these circuits. The slapping automatism, when viewed in this light, may represent a behavioral expression of underlying prefrontal dysregulation, shared by both seizure propagation and obsessive-compulsive symptoms.

### 2.3 Clinical implications and surgical strategy

This case series has direct clinical implications for the pre-surgical evaluation and planning in patients with focal epilepsy, especially those presenting with slapping or clapping automatisms. While such automatisms may be superficially classified as “motor” features with presumed frontal origin, our SEEG data emphasize the need for careful multimodal assessment, including consideration of temporal onset with frontal propagation.

Notably, all five patients underwent tailored resections or thermocoagulation informed by SEEG-defined epileptogenic networks. Three patients achieved Engel Class I outcomes, and two achieved Class II, confirming the effectiveness of network-based surgical interventions. Additionally, the observed improvement in psychiatric symptoms following surgery in Case 5 encourages a broader perspective on surgical goals—not just seizure freedom, but also the amelioration of comorbid neuropsychiatric dysfunction.

### 2.4 Limitations and future directions

This study is limited by its small sample size, short follow-up duration and retrospective design. Future research should investigate the prevalence and electrophysiological correlates of slapping automatisms in larger cohorts, ideally integrating quantitative analysis of SEEG dynamics, functional connectivity, and longitudinal psychiatric assessments.

## 3 Conclusion

In summary, our study provides novel electrophysiological evidence linking slapping automatisms to prefrontal seizure propagation, particularly involving the orbitofrontal and dorsolateral prefrontal cortices. We further demonstrate that shared pathological circuits underlie both frontal-temporal epilepsy and OCD, suggesting a unified frontotemporal-subcortical network responsible for motor automatisms and compulsive behaviors. These insights expand our understanding of seizure semiology and psychiatric comorbidities and support a network-targeted approach to epilepsy surgery.

## Data Availability

The original contributions presented in the study are included in the article/Supplementary material, further inquiries can be directed to the corresponding authors.
