# Supplementary material for: Slapping automatism in epileptic seizures: a case series
Source: Front Hum Neurosci. 2025 Aug 25;19:1593597. doi: 10.3389/fnhum.2025.1593597 (PMC12415034; doi:10.3389/fnhum.2025.1593597)
Supplement: Supplementary file 1 [file Supplementary_file_1.docx]

Supplementary Material

**Supplementary table 1.** Pt: patient; M: male; F: female; y: year; m: month; R right; L: left; H: hippocampus; T: temporal lobe; F: frontal lobe. Abbreviations: ATL = anterior temporal lobectomy; HC = hippocampus; AMY = amygdala; AI = anterior insula; FO = frontal operculum; OF = orbital frontal; OFG = orbital frontal gyrus; TPO junction = temporo-parieto-occipital junction; VMPFC = ventromedial prefrontal cortex; RF-TC = radiofrequency thermocoagulation.

**Supplementary table 2.** Pt, Patient; MMSE, Minimum Mental State Examination (maximum score: 30); MOCA, Montreal Cognitive Assessment (maximum score: 30); HAMD, Hamilton Depression Scale (maximum score: 30); HAMA, Hamilton Anxiety Scale (maximum score: 56); Y-BOCS, Yale-Brown Obsessive Compulsive Scale (maximum score: 40). /: missing values for the patients. Patient 5 could not complete the assessment because of severe cognitive impairment.

**Supplementary figure 1.** Clinical information related to patient 4. **A:** Scalp EEG: the upper panel shows interictal EEG, and the lower panel shows ictal EEG. Bandpass filter: 0.5–30z; Sensitivity: 10 μV/mm. **B:** hand slapping behavior observed during the seizure. **C:** MRI, patchy abnormal signal lesions in the bilateral frontoparietal lobes. **D:** PET, hypometabolism in the left frontal lobe. **E:** Coregistered preoperative MRI and post-implantation SEEG CT, with the red circle indicating the frontal electrode related to hand slapping. **F:** Postoperative MRI, with the red circle marking the resected brain region. **G:** Ictal SEEG, the blue channels indicate the SOZ, and both the blue and red channels exhibit discharge evolution during hand slapping. The red arrow indicates interictal epileptic discharges, the red triangle marks the seizure onset, and the red dashed line represents the onset of the hand slapping symptom. The scale marker is located in the upper left corner of the EEG. Bandpass filter: 0.5–70z; Sensitivity: 70μV/mm. **H:** Timeline diagram of SEEG discharges and clinical symptoms. Abbreviations: LaPFC, left anterior prefrontal cortex; LdACC, left dorsal anterior cingulate cortex; LVLPFC, left ventrolateral prefrontal cortex; LOG, lateral orbitofrontal gyrus.

**Supplementary figure 2.** Clinical information related to patient 4. **A:** Scalp EEG: the upper panel shows interictal EEG, and the lower panel shows ictal EEG. Bandpass filter: 0.5–30z; Sensitivity: 10 μV/mm. **B:** hand slapping behavior observed during the seizure. **C:** MRI, scattered small patchy abnormal signal lesions in the bilateral frontoparietal lobes and atrophy of the bilateral hippocampi. **D:** PET, hypometabolism in the bilateral frontal and parietal lobes. **E:** Coregistered preoperative MRI and post-implantation SEEG CT, with the red circle indicating frontal electrodes exhibiting interictal epileptiform discharges. **F:** Postoperative MRI, with the red circle marking the resected brain region. **G:** Iinterictal SEEG, the red and blue channels indicate frontal brain regions with interictal epileptiform discharges. The red arrow indicates interictal epileptic discharges, the red triangle marks the seizure onset. The scale marker is located in the upper left corner of the EEG. Bandpass filter: 0.5–30z; Sensitivity: 70μV/mm.
